# Neural Responses to Rapid Facial Expressions of Fear and Surprise

**DOI:** 10.3389/fpsyg.2017.00761

**Published:** 2017-05-10

**Authors:** Ke Zhao, Jia Zhao, Ming Zhang, Qian Cui, Xiaolan Fu

**Affiliations:** ^1^State Key Laboratory of Brain and Cognitive Science, Institute of Psychology, Chinese Academy of SciencesBeijing, China; ^2^Key Laboratory of Mental Health, Institute of Psychology, Chinese Academy of SciencesBeijing, China; ^3^Department of Psychology, University of Chinese Academy of SciencesBeijing, China; ^4^Key Laboratory of Cognition and Personality (Ministry of Education) and Faculty of Psychology, Southwest UniversityChongqing, China; ^5^Department of Psychology, Dalian Medical UniversityDalian, China

**Keywords:** fearful face, surprised face, amygdala, recognition

## Abstract

Facial expression recognition is mediated by a distributed neural system in humans that involves multiple, bilateral regions. There are six basic facial expressions that may be recognized in humans (fear, sadness, surprise, happiness, anger, and disgust); however, fearful faces and surprised faces are easily confused in rapid presentation. The functional organization of the facial expression recognition system embodies a distinction between these two emotions, which is investigated in the present study. A core system that includes the right parahippocampal gyrus (BA 30), fusiform gyrus, and amygdala mediates the visual recognition of fear and surprise. We found that fearful faces evoked greater activity in the left precuneus, middle temporal gyrus (MTG), middle frontal gyrus, and right lingual gyrus, whereas surprised faces were associated with greater activity in the right postcentral gyrus and left posterior insula. These findings indicate the importance of common and separate mechanisms of the neural activation that underlies the recognition of fearful and surprised faces.

## Introduction

Different emotions are associated with specific facial expressions, and the recognition of these facial expressions is important for social communication (Haxby et al., 2002). Among the six basic facial expressions (fear, sadness, surprise, happiness, anger, and disgust), fear and surprise are easily confused because surprised and fearful faces are “wide-eyed, information gathering” facial expressions (Kim et al., 2003, 2004; Zhao et al., 2013). A fearful expression involves open eyes and mouth and conveys shock in response to a frightening event, which signals a potential threat. A surprised expression also involves wide eyes and an open mouth, which indicate unexpectedness and novelty (Schroeder et al., 2004; Duan et al., 2010). According to Ekman’s (1993) terminology, surprise is expressed by specific combinations involving two, three, or four action units, including the raised inner and outer brow, the raised upper eyelid, and the open mouth. Fear patterns also involve these action units; however, two specific action units, namely, the brow lower and the lip stretcher, might be part of fear patterns but not of surprise patterns (Ekman, 1993).

The recognition of facial expression is mediated by a distributed neural system (Haxby et al., 2000; Adolphs, 2002). This process is associated with increased activation in numerous visual areas (fusiform gyrus and lingual gyrus), temporal areas (middle/superior temporal gyrus and MTG), prefrontal areas (medial frontal gyrus and middle frontal gyrus), and limbic areas (amygdala and parahippocampal gyrus).

The discrimination of fear and surprise may be reflected in the brain activity patterns that underlie facial expression recognition. A fear expression indicates a potential threat, whereas surprise conveys a sense of novelty or unexpectedness (Adolphs et al., 1995; Schroeder et al., 2004). Fear has been described as negatively valenced surprise (Vrticka et al., 2014). Although no studies have directly investigated the different neural mechanisms that underlie these two faces, some brain regions have been found to be specialized for different emotional functions. The parahippocampal gyrus has been found to exhibit greater activation for surprised faces than fearful faces because surprised faces are consciously or unconsciously perceived due to their novelty (Schroeder et al., 2004; Duan et al., 2010). Correspondingly, the conscious and unconscious perception of faces with fearful expressions has been found to be associated with a significant amygdala response, which suggests a role of vigilance and the close monitoring of environmental cues (Morris et al., 1996; Whalen et al., 1998). However, other studies provide evidence that the human amygdala is also responsive to surprised facial expressions (Kim et al., 2003; Kim et al., 2004). A recent study revealed that poorer classification accuracy among all emotion categories was observed in the amygdala and hippocampus (Saarimaki et al., 2016).

As mentioned above, the specific brain regions that are most sensitive to fear or surprise remain unknown. To investigate the specific neural substrates, we directly contrasted the neural responses to fearful faces and surprised faces. In addition, previous studies have reported extremely high accuracies in the recognition of different emotions; however, the presentation times in these studies are long (Duan et al., 2010; Saarimaki et al., 2016). In a previous study, we found that performance in recognizing fearful and surprised faces was lower when the presentation time of the target face was short (100–500 ms) (Zhao et al., 2013). The present study used event-related functional magnetic resonance imaging (fMRI) to identify the neural substrates that mediate the perception of rapid surprised and fearful faces in healthy volunteers. By comparing the different patterns of neural activity in response to these faces, we identified similarities and differences between the mechanisms that underlie the recognition of fearful and surprised facial expressions.

## Materials and Methods

### Subjects

Fifteen healthy subjects (8 males) aged 20.5 ± 1.24 years were recruited for the experiment. All of the subjects were right-handed, free of neurological or psychiatric diseases, and had normal or corrected-to-normal vision. The subjects were paid for their participation. The experimental procedures were approved by the IRB of the Faculty of Psychology, Southwest University, and informed written consent was obtained from all of the subjects.

### Stimulation and Experimental Design

**FIGURE 1:**
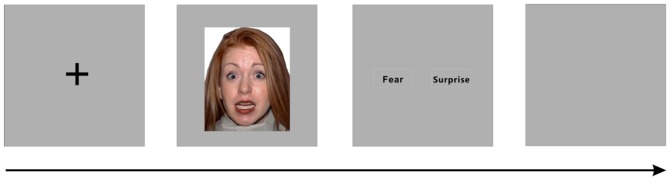
**Illustration of a single trial of facial expression recognition**.

The present study investigated the perception of surprised and fearful faces. The target stimuli included images of two types of facial expressions (fear and surprise) posed by 43 individual human models from the NimStim database (Tottenham et al., 2009). Eighty-six images were selected from the database and trimmed to 192 × 220 pixels. The protocol was based on Ekman and Friesen’s Brief Affect Recognition Test (Ekman and Friesen, 1974). In each trial, a black fixation cross was initially presented in the center of the silver–gray background for 200 ms, followed by a facial expression image presented in the center of the screen for 100, 300, or 500 ms. The subjects were instructed to identify the facial expression by using the right thumb to press a key (“1” or “2”). After the participants selected an answer, an inter-trial interval (ISI) was randomly inserted between the trials (**Figure 1**). The entire trial lasted 6 s, and the ISI did not include the fixation presentation, face presentation, and response time. We also included four blank intervals of 6 s duration among the trials.

### Data Acquisition and Analysis

Functional magnetic resonance imaging data were acquired using a Siemens 3.0 Tesla Trio scanner with a standard head coil at the Key Laboratory of Cognition and Personality (Ministry of Education) at Southwest University (China). The functional scanning used a whole-brain gradient-echo, echo-planar-imaging sequence, and the repetition time was 2000 ms (30 ms echo time, 32 slices, 3.44 mm × 3.44 mm in-plane resolution, 1 mm slice gap, voxel size 3.4 × 3.4 × 4, field of view 220 mm × 220 mm, matrix 64 × 64, and flip angle = 90°).

Complete fMRI data were acquired for 15 subjects and included in the following analysis. The data were preprocessed and analyzed using Statistical Parametric Mapping software SPM8 (Wellcome Trust Center for Neuroimaging, London, UK). The first five volumes for each subject were discarded to allow for signal equilibration. The images were slice-time corrected, motion corrected, normalized to the Montreal Neurological Institute (MNI) space at 3 mm × 3 mm × 3 mm, and spatially smoothed using a Gaussian kernel of 8 mm full width at half maximum (FWHM) (Ashburner and Friston, 2005). Then, two types of individual events (time-locked to the photographs) were modeled by a canonical hemodynamic response with two conditions: facial expressions of fear and surprise. A general linear model (GLM) was applied to the data to estimate the parameters of event-related activity corresponding to correct trials for each voxel in the volume under two conditions. Incorrect trials of both fearful faces and surprised faces were modeled separately in the GLM and discarded in the following analyses. Finally, statistical parametric maps with *t*-values were generated for each condition and each subject after first-level analysis (Calhoun et al., 2004).

A second-level random effects approach was applied to the group-level statistical analyses, which estimated the error variance of the interested conditions across subjects. During the second-level analysis, *t*-tests and conjunction analysis were applied to the two condition to identify the brain activations under each condition and the common activations of the two conditions, respectively. To examine the brain regions that are particularly involved in the perception of a specific emotional expression, the two emotional conditions were directly compared using paired *t*-tests (surprise vs. fear, fear vs. surprise). Multiple comparisons were applied to the inferences from the statistical parametric maps for the threshold corrected across gray matter in whole brain with Monte Carlo simulations (the cluster connection radius was 5 mm, and the number of Monte Carlo simulations was set to 1000) (Forman et al., 1995). The mask we used in the multiple correction with Monte Carlo simulations was extracted from WFU_PickAtlas software (gray matter in tissue type) (Maldjian et al., 2003) and then resampled to 3 × 3 × 3 volume as the gray matter mask (the number of voxels in the mask was 19956).

In addition, a correlation analysis was utilized to assess the associations between the subject’s sensitivity and brain activation under the two experimental conditions. The correlations between the sensitivity index (*d*′) and the brain activations of each subject for each condition were calculated. The common areas that were significantly correlated with the recognition score under the two face stimuli were extracted as regions of interest (ROIs) using the MarsBar toolbox^1^. Then, the brain activities in the constructed ROIs were analyzed.

## Results

**FIGURE 2:**
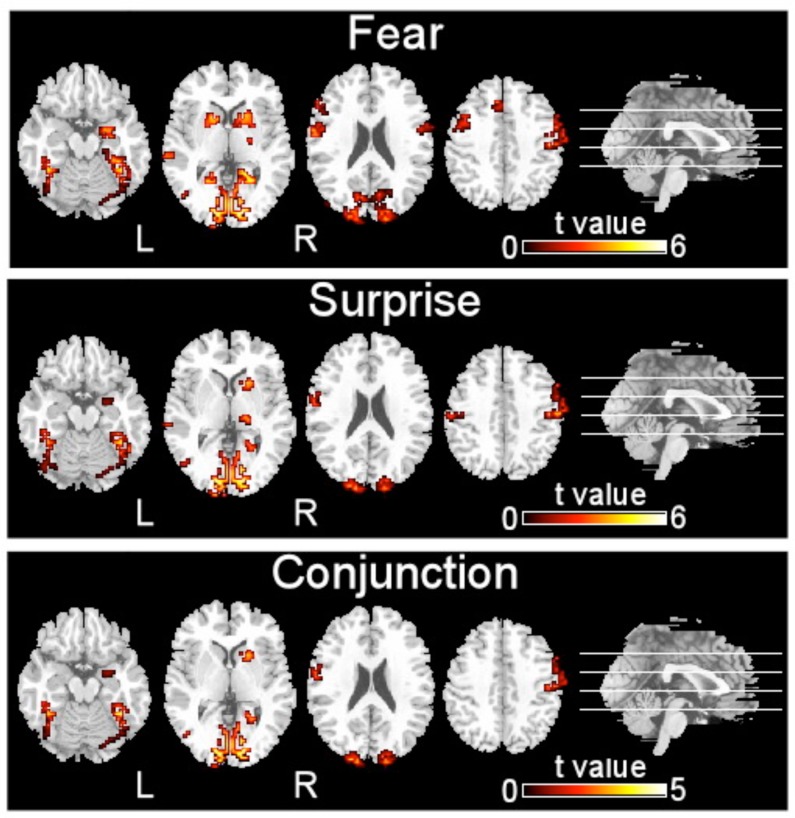
**Brain regions activated by two types of facial expressions, fear and surprise (*p* < 0.001, corrected with Monte Carlo simulations)**.

**FIGURE 3:**
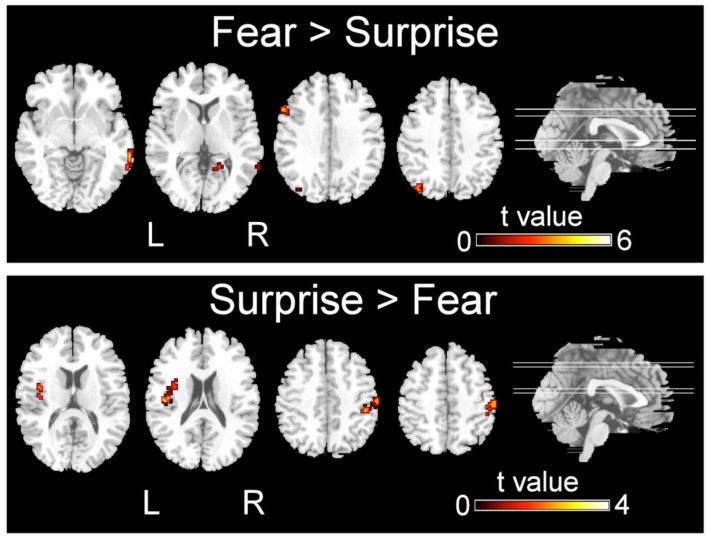
**Significant differences in the activation of brain regions during the recognition of fearful versus surprised faces (*p* < 0.001, corrected with Monte Carlo simulations)**.

There was no difference in recognition accuracy scores between fearful faces (0.78 ± 0.08) and surprised faces (0.77 ± 0.11; *t* = 0.52, *p* = 0.61). We initially determined the brain regions that exhibited increased activation when the subjects watched the two types of facial expressions (**Figures 2**, **3**). To illustrate the detailed activation information, the MNI coordinates of the peak *T*-values and voxel numbers for all significant clusters were extracted and are displayed in **Tables 1**–**5**.

**Table 1:** Neural activity in response to facial expression of fear.

Brain region	MNI co-ordinates *x, y, z*	Volume (voxels)
L middle temporal gyrus	–51, –76,10	52
L putamen	–18,11,4	43
L inferior occipital gyrus	–42, –79, –11	26
L postcentral gyrus	–60, –7,16	117
L cuneus	–15, –91,1	480
L precentral gyrus	–42,8,31	132
L supplementary motor area	–6,14,52	79
R precentral gyrus	57, –10,49	332
R inferior occipital gyrus	39, –79, –8	30
R amygdala	27, –7, –14	203
R parahippocampal gyrus	18, –49, –5	539


*L = left, R = Right. Significant at corrected *p* < 0.001.*

**Table 2:** Neural activity in response to facial expression of surprise.

Brain region	MNI co-ordinates *x, y, z*	volume (voxels)
L middle occipital gyrus	–15, –103,10	360
L supplementary motor area	–3,11,55	38
L postcentral gyrus	–57, –16,31	114
R lentiform nucleus	21,14,4	64
R inferior occipital gyrus	39, –79, –8	29
R calcarine	3, –82,1	440
R postcentral gyrus	57, –16,52	241
R parahippocampal gyrus/amygdala	27, –7, –14	20
R precentral gyrus	54, –7,10	19


*L = left, R = Right. Significant at corrected *p* < 0.001.*

**Table 3:** Conjunction of neural activity for facial expressions of fear and surprise.

Brain region	MNI co-ordinates *x, y, z*	volume (voxels)
L middle occipital gyrus	–15, –103,7	97
L fusiform	–42, –43, –23	38
L inferior occipital gyrus	–42, –79, –11	26
L postcentral gyrus	–60, –13,28	64
L cuneus	–15, –91,1	162
L supplementary motor area	–3,11,55	37
R postcentral gyrus	57, –16,52	199
R inferior occipital gyrus	39, –79, –8	29
R calcarine	3, –82,1	416
R putamen	21,14,4	62
R parahippocampal gyrus (amygdala)	27, –7, –14	20


*L = left, R = Right. Significnat at corrected *p* < 0.001.*

**Table 4:** Neural activity showing more activation for fear than for surprise.

Brain region	MNI co-ordinates x, y, z	volume (voxels)
L precuneus	–39, –73,37	14
L middle frontal gyrus	–57,17,34	10
R middle temporal gyrus	63, –40, –14	36
R lingual gyrus	18, –49,1	12


*L = left, R = Right. Significant at corrected *p* < 0.001.*

**Table 5:** Neural activity showing more activation for surprise than for fear.

Brain region	MNI co-ordinates *x, y, z*	volume (voxels)
L insula	–45, –19,19	29
R postcentral gyrus	42, –34,34	47


*L = left, R = Right. Significant at corrected *p* < 0.001.*

The brain regions that exhibited increased activation in response to fearful faces included the left postcentral gyrus, left middle temporal gyrus, left cuneus, left putamen, left inferior occipital gyrus, left precentral gyrus, left supplementary motor area, right precentral gyrus, right inferior occipital gyrus, right parahippocampal gyrus, and right amygdala (*p* < 0.001, corrected with Monte Carlo simulations; **Figure 2** and **Table 1**). Compared to fearful faces, surprised faces were associated with increased activation of the left postcentral gyrus, left middle occipital gyrus, left supplementary motor area, right lentiform nucleus, right calcarine, right postcentral gyrus, right precentral gyrus, right inferior occipital gyrus, right parahippocampal gyrus, and right amygdala (*p* < 0.001, corrected; **Figure 2** and **Table 2**).

The conjunction analysis revealed that the brain regions that exhibited increased activation in response to both the surprised and fearful faces included the left postcentral gyrus, left middle occipital gyrus, left fusiform, left inferior occipital gyrus, left cuneus, left supplementary motor area, right postcentral gyrus, right inferior occipital gyrus, right calcarine, right putamen, right parahippocampal gyrus, and right amygdala (**Figure 2**).

Regarding the differences in the perceptual processing of fearful faces versus surprised faces, the significant clusters included the left precuneus, left middle frontal gyrus, right MTG and right lingual gyrus for the contrast between the fear and surprise conditions (*p* < 0.001, corrected; **Figure 3** and **Table 4**). For the contrast between the surprise and fear conditions, differences were located at two clusters, including the left posterior insula and right postcentral gyrus (*p* < 0.001, corrected; **Figure 3** and **Table 5**).

**FIGURE 4:**
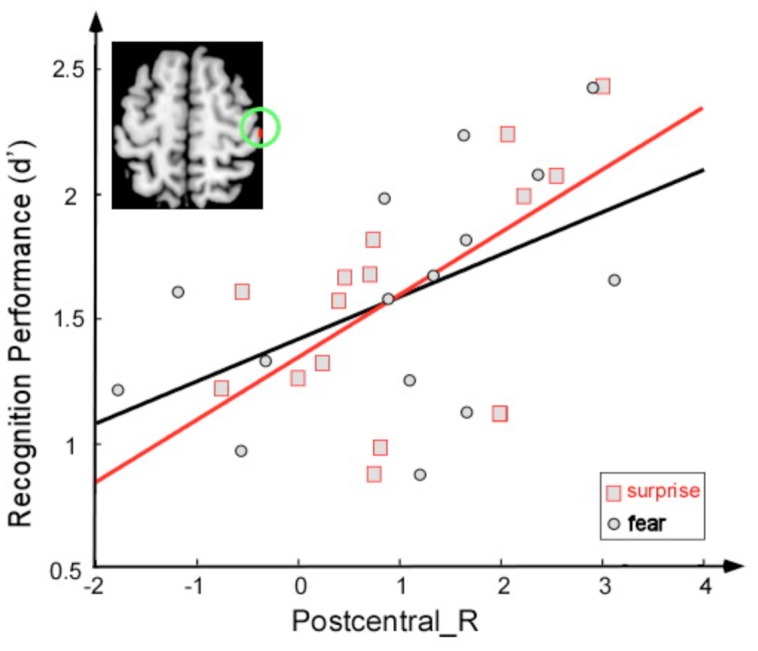
**Correlation between the sensitivity index (*d*′) and activation magnitude (*T*-value) under two conditions: fearful face stimuli (black) and surprised face stimuli (red)**.

Correlation analyses were employed to examine the relationship between sensitivity of discrimination between two faces (a score calculated as the *Z* score for a correct response minus the *Z* score for a false alarm) and brain activity (**Figure 4**). The activity of the right postcentral area was significantly correlated with this sensitivity index under the fearful face condition (*r* = 0.52, *p* < 0.05) and under the surprised face condition (*r* = 0.61, *p* < 0.05).

## Discussion

The current findings indicated similarities and differences in the neural mechanisms that underlie the recognition of fearful and surprised faces. In the present study, brain regions within the temporal and occipital cortices, such as the left fusiform gyrus, were activated during the perception of fearful and surprised faces, which may indicate these brain regions are involved in the general perceptual recognition of facial expressions (Haxby et al., 2000; Winston et al., 2004). Regions of the occipital and temporal visual cortices play a critical role in the perceptual processing of socially and emotionally relevant visual stimuli (Haxby et al., 2000, 2002). Increased activation of these areas may represent top-down modulatory effects on the visual processing stream, which reflect attentional enhancement as a result of emotional significance (Vuilleumier and Schwartz, 2001; Pessoa et al., 2002). In addition, fearful faces appear sufficient to evoke increased amygdala activation. Our results indicate that the amygdala (particularly in the right hemisphere) is responsive to surprised faces and are consistent with a previous study reporting that the right amygdala was activated in response to both fearful and surprised faces (Kim et al., 2003). The right parahippocampal gyrus was similarly activated during the recognition of fearful and surprised faces. The amygdala and hippocampus are strongly interconnected and receive inputs from extrastriate visual areas in the occipital and temporal cortices (Amaral and Insausti, 1992; Morris et al., 1999). Our findings indicated that the amygdala and parahippocampal gyrus form an important part of the emotion network but are unable to distinguish between fearful and surprised faces. This result is consistent with a previous study that found that although limbic regions, including the amygdala, hippocampus, and thalamus, appear to form an important part of the emotion network, the limbic components of the network revealed poorer classification accuracy than did the cortical components (Vrticka et al., 2014).

Our results indicate that fearful faces induced more activation than did surprised faces in the frontal and temporal lobes. The middle frontal gyrus was activated during fearful face recognition. Previous research has indicated that this brain region is implicated in contingency awareness in human aversive conditioning (Knight et al., 2004; Carter et al., 2006). The ‘attentional network’ has been extensively researched and is thought to involve fronto-parietal regions, including the middle frontal gyrus (MFG) (Pessoa, 2009). Thus, the activity of this region may reflect the attention being paid to fearful faces. Neurons in the human MTG respond to socially important aspects of faces such as expression, orientation, and eye-gaze direction (Perrett et al., 1985; Hasselmo et al., 1989). In a study by Morris et al. (1998), the right MTG received a greater contribution from the amygdala during the processing of fearful expressions (Morris et al., 1998). Depth EEG results have indicated that the amygdala is activated along with the MTG (Krolak-Salmon et al., 2004). A previous study identified the activation of this region during the recognition of fear versus disgust (Phillips et al., 1998). In other work, functional activation specifically associated with a fearful face prime was found in the activated bilateral middle temporal gyrus (Fan et al., 2011). In addition, anomia for facial emotions has been reported in patients with lesions in the right middle temporal gyrus (Rapcsak et al., 1993; Cornwell et al., 2008). The activation of this brain region might be due to the reception and correct labeling of potential threat information from fearful faces.

The facial expression of surprise has a distinct character and might be universally recognized. Psychological theories suggest that surprise is an adaptive mechanism to restructure and extend cognitive concepts following the analysis of an unexpected event (Schutzwohl, 1998); moreover, it provides important indicators of emotion with respect to unexpectedness and novelty (Schroeder et al., 2004). In the present study, surprised faces induced greater activation in the postcentral cortices than did fearful faces, which suggests that additional activity in this region was required to correctly recognize surprised faces. The sensitivity of recognition between two faces was positively correlated with the activation of this area for both the fearful face and surprised face conditions. One interpretation of these findings is that viewing facial expressions of emotion triggers an emotional response in the perceiver that mirrors the emotion presented in the stimulus (Pitcher et al., 2008; Wood et al., 2016). Moreover, the representation of this emotional response in the somatosensory cortices may provide information regarding the emotion. In particular, the somatosensory, motor, and premotor cortices have been associated with emotion recognition in research with lesion patients (Adolphs et al., 2000) and research using transcranial magnetic stimulation (TMS) (Pourtois et al., 2004; Pitcher et al., 2008). Regarding the posterior insula, previous studies have suggested that the left and right insula preferentially encode positive and negative affect, respectively (Craig, 2009). Left insular activation has been identified in subjects experiencing joy (Takahashi et al., 2008). Damage to this area may impair gustatory information processing (Calder et al., 2001). Thus, the greater activation of this brain region in the surprise condition might be attributed to the surprised face being experienced as more positive than the fearful face. Fear was described as negatively valenced surprise in a recent study (Vrticka et al., 2014).

## Conclusion

The present study used fMRI to explore the activation of different brain regions in response to fearful and surprised faces. Our results indicate that the limbic system, including the amygdala and parahippocampal gyrus, is responsible for both of these faces. The fearful faces elicited greater activation in some frontal regions and the right middle temporal gyrus, whereas the insula and postcentral cortices were largely activated in the recognition of surprised faces. These results suggest that fear leads to greater activation of the attention and memory systems, whereas surprise results in greater activation of the emotion experience system.

## Ethics Statement

The experimental procedures were approved by the local ethics committee in Southwest University (China).

## Author Contributions

KZ, JZ, XF contributed in designing the experiment, analyzing the data, and writing the manuscript. MZ contributed in collecting the data and analyzing the data, and QC contributed in writing the manuscript.

## Conflict of Interest Statement

The authors declare that the research was conducted in the absence of any commercial or financial relationships that could be construed as a potential conflict of interest.

## Footnotes

1 http://marsbar.sourceforge.net
